# Gut microbiota signatures in tuberous sclerosis complex and epilepsy: a pilot study

**DOI:** 10.3389/fnins.2025.1655456

**Published:** 2025-11-18

**Authors:** Emerenziana Ottaviano, Matteo Domenico Marsiglia, Camilla Ceccarani, Silvia Ancona, Francesca Triva, Francesca La Briola, Stefania Bergamoni, Federica Teutonico, Alice Pompili, Ilaria Viganò, Emilia Ricci, Aglaia Vignoli, Elisa Borghi

**Affiliations:** 1 Department of Health Sciences, Università Degli Studi di Milano, Milan, Italy; 2 Institute of Biomedical Technologies, National Research Council, Milan, Italy; 3 Child Neurology and Epilepsy Centre, ASST Santi Paolo e Carlo, Milan, Italy; 4 Childhood and Adolescence Neurology and Psychiatry Unit, ASST GOM Niguarda, Milan, Italy; 5 Università Degli Studi di Milano, Milan, Italy

**Keywords:** tuberous sclerosis complex, gut microbiota brain axis, epilepsy, inflammation, children

## Abstract

**Objective:**

Tuberous sclerosis complex (TSC) presents with a broad clinical spectrum. While some individuals exhibit mild symptoms, most experience seizures and neuropsychiatric comorbidities. Emerging evidence suggests that both genetic and environmental factors, including gut microbiota, may influence epilepsy susceptibility. The microbiota–gut–brain axis (MGBA) is a key communication pathway through which intestinal microbes impact the central nervous system. Although the role of the MGBA in the pathogenesis of neurological diseases, particularly seizures, has been explored in both animal models and humans, data specific to TSC are lacking.

**Methods:**

In this exploratory study, we assessed whether individuals with TSC (*n* = 15) display a distinct gut microbial signature using V3–V4 16S rRNA sequencing. Their profiles were compared with two control groups: 18 children with epilepsy (EPI) and 12 age- and sex-matched healthy controls (HC). Stool short-chain fatty acid (SCFA) levels and dietary intake were also evaluated.

**Results:**

No significant differences were observed among the three groups in dietary intake, SCFA and branched-chain fatty acid (BCFA) levels, or alpha-diversity. Beta-diversity analysis showed a non-significant trend toward clustering of TSC and EPI samples, indicating a shared microbial profile distinct from HC. Taxonomic analysis revealed a reduction in Firmicutes—particularly the *Ruminococcaceae* family and the genus *Gemmiger*—in both TSC and EPI groups, consistent with epilepsy-associated dysbiosis. Notably, the TSC group showed a specific enrichment in *Akkermansiaceae*, a feature also reported in other neurodevelopmental disorders such as CDKL5 deficiency disorder and cerebral palsy.

**Significance:**

These preliminary findings suggest that gut microbiota alterations may contribute to neuroinflammatory processes linked to epileptogenesis and comorbidities in TSC. Further studies are needed to validate these results and explore microbiota-based therapeutic strategies aimed at improving outcomes and quality of life for individuals with TSC and their caregivers.

## Highlights

- This is the first study to investigate the possible role of gut microbial communities in Tuberous Sclerosis Complex (TSC), with a specific focus on epilepsy
- We compared the gut microbiota profile of patients with TSC with those of subjects with epilepsy and healthy controls
- Both TSC and epilepsy groups showed a depletion in *Gemmiger* and *Faecalibacterium*, taxa associated with anti-inflammatory functions and butyrate production.
- While many microbial alterations were shared, the TSC group showed increased *Akkermansiaceae*, a genus implicated in other neurological disorders.
- These preliminary results highlight the need for larger studies to confirm findings and clarify the microbiota-gut-brain axis role in TSC.

## 1 Introduction

Tuberous sclerosis (TSC) is a rare genetic, autosomal-dominant, multisystem disease (Crino et al., 2006), with an incidence of approximately 1 case per 6,000–10,000 live births (Henske et al., 2016).

TSC is caused by mutations in the *TSC1* or *TSC2* genes encoding for hamartin and tuberin, respectively. These proteins form a complex that regulates the mammalian/mechanistic target of rapamycin (mTOR), and the hyperactivation of mTOR, caused by loss of *TSC1* or *TSC2,* is involved in the formation of benign tumors. Therefore, patients with TSC (pwTSC; Laplante and Sabatini, 2012) may develop benign tumors in several organs, including kidneys (Neumann et al., 1998), lungs (Vicente et al., 2004), heart (Jóźwiak et al., 2005), and central nervous system (CNS; Gomez et al., 1999). Lesions in the CNS, such as cortical tubers and subependymal astrocytomas, may cause intellectual disability (ID) and behavioral disorders. Indeed, most individuals exhibit tuberous-sclerosis-associated neuropsychiatric disorders (TANDs) during their lives, and children have an increased risk of developing Autism Spectrum Disorder (ASD; Sparagana and Roach, 2000). TSC is associated with epilepsy in 70–90% of patients, frequently manifesting with medication-resistant seizures (White et al., 2001; Chu-shore et al., 2010; Curatolo et al., 2015; Vignoli et al., 2013). While seizures are generally thought to originate from cortical tubers, marked by dysmorphic neurons and giant cells, the exact mechanism of epileptogenesis in TSC remains complex and not fully understood (Rastin et al., 2023).

The microbiota-gut–brain axis (MGBA) has recently been widely investigated in the etiopathogenesis of epilepsy, both in animal models and human studies (Zhang et al., 2025; da Silva et al., 2025; Ceccarani et al., 2021; Riva et al., 2025). Intestinal microorganisms may contribute to seizure onset and medication resistance through various mechanisms, including the promotion of a basal inflammatory state (Zhao et al., 2023), altering gastrointestinal barrier homeostasis, and the production of a wide range of bioactive metabolites (Wells et al., 2017). Gut bacteria produce both neuroactive compounds, such as tryptophan, serotonin, and dopamine, which can reach the central nervous system through the bloodstream or influence neurons in the enteric nervous system (Stilling et al., 2014) and other relevant metabolites such as short-chain fatty acids (SCFAs; Wells et al., 2017). These latter, particularly butyric acid, exert diverse effects that may be relevant to epilepsy, including epigenetic modulation, neuroprotection, and both local and systemic anti-inflammatory actions (Kalkan et al., 2025). By influencing neuronal excitability, synaptic plasticity, and inflammatory pathways, SCFAs could contribute to seizure mitigation and improved neurological outcomes (Yan et al., 2025).

Since pathogenic variants alone cannot account for the broad spectrum of clinical manifestations in pwTSC, in this pilot study, we investigated whether the gut microbiota might contribute to the severity of comorbidities, particularly seizure occurrence, through the gut-brain axis.

## 2 Materials and methods

### 2.1 Cohort enrollment

We enrolled individuals who had been diagnosed with TSC disorder at the Department of Child Neuropsychiatry of the ASST Santi Paolo e Carlo and of the GOM Niguarda. As a control group, we included healthy controls, age- and sex-matched, and subjects with drug-susceptible (DSE) or drug-resistant epilepsy (DRE). We excluded individuals who had used antibiotics or probiotics within 1 month before the study, and subjects who presented metabolic diseases or infectious diseases at enrollment. The study was approved by the Local Ethics Committee (protocol number 2016/ST/199, 28 July 2016). Written informed consent was obtained from parents and/or legal guardians of the enrolled patients/healthy subjects.

Caregivers were asked to fill out a 3-day dietary survey. The diary included three consecutive days, one of which was during the weekend. Dietary food records were processed using a commercially available software (MètaDieta, METEDA srl, Italy). Anthropometric evaluation completed the nutritional survey. The stool transition time was estimated by the Bristol Stool Form Scale (BSFS; Lewis and Heaton, 1997).

### 2.2 Fecal short-chain fatty acid quantification

SCFAs were extracted by homogenizing 200 mg of stool in 1 mL of water. From 300 μL of homogenate, 700 μL of water, 200 μL of orthophosphoric acid (85%), and 100 μL of internal standard (2-ethylbutyric acid, 20 mM) were added. The mixture was extracted with 500 μL diethyl ether/heptane (1:1) and centrifuged for 5 min to recover the organic phase. Acetic, propionic, isobutyric, butyric, and isovaleric acids were quantified by GC–MS (GC 8860 System-MSD 5977C, Agilent) using a DB-WAX Ultra Inert column. Compound identity was verified with pure standards by comparing retention times and MS spectra. Calibration standards (5–0.3125 mM) were extracted alongside samples using 2-ethylbutyric acid as the internal standard. Data were processed with MassHunter software (Agilent).

### 2.3 Microbial DNA extraction and 16S rRNA gene sequencing of human gut microbiota

Bacterial genomic DNA from stool samples was extracted using the Spin Stool DNA Kit (Stratec Molecular, Berlin, Germany) as described by Di Fede et al. (2021). DNA concentration was measured with the DNA High Sensitivity Qubit kit (ThermoFisher Scientific, Waltham, MA, United States). The V3–V4 regions of the bacterial 16S rRNA gene were sequenced by Macrogen (Seoul, Republic of Korea) following the Illumina 16S Metagenomic Sequencing Library Preparation protocol (Illumina, San Diego, CA, United States).

Amplicon sequence variants (ASVs) were identified using the DADA2 pipeline (v1.18.0) for read filtering, trimming, and denoising, and downstream analyses were conducted in R with the phyloseq package (v1.34.0) and custom scripts (Callahan et al., 2016). Alpha diversity was assessed using Chao1, Shannon, Observed species, and Faith’s PD metrics, while beta diversity was analyzed with weighted and unweighted UniFrac distances and visualized by PCoA (Lozupone et al., 2011). Taxonomic assignment was performed using the 8-mer classifier of the RDP database (release 11.5; Wang et al., 2007) and the GTDB 16S rRNA database (release r207; Parks et al., 2022).

### 2.4 Statistical analysis

Non-categorical variables were expressed as mean ± SD, and relative abundances as percentages. Group comparisons for alpha- and beta-diversity and taxonomic data were performed using the Kruskal–Wallis test with Dunn’s post-hoc correction. Co-abundance matrices were generated using Pearson’s correlation and Ward’s hierarchical clustering. Integration of diet, fatty acids, and microbial genera was conducted via sparse discriminant analysis using a classic PLS algorithm. Unless otherwise stated, *p*-values were Benjamini–Hochberg adjusted, with significance set at *p* < 0.05.

## 3 Results

### 3.1 Cohort description

We enrolled 15 individuals with TSC (“TSC” group; mean age 8.2 ± 5.5, 8 males), including 13 with pathogenic variants in *TSC2* and 1 with a pathogenic variant in *TSC1*. In one patient, no pathogenic variant was identified. Among individuals with TSC, 6 had drug-resistant seizures, while in 8 patients, seizures were under control. All patients with DRE were on 2–3 anti-seizure medications (ASMs), and patients with DSE were on monotherapy resulting in a total of 14/15 (93%) patients receiving ASMs. One TSC individual did not experience epilepsy. None of the pwTSC included in the study were on everolimus. Besides epilepsy, 7 individuals showed ID, 4 ASD, and 1 attention-deficit/hyperactivity disorder (ADHD).

As control groups, we collected stool samples from 12 healthy controls (“HC” group; mean age 9.1 ± 4.6, 5 males) and 18 subjects with epilepsy (“EPI” group; mean age 12.9 ± 6.0, 8 males), 8 with DRE and 10 with DSE, all undergoing ASMs. Among children with DRE, 3 had ID and 1 ASD; no child with DSE presented with ID or neurodevelopmental disorder. Due to the small cohort, in the analyses we did not divide TSC and EPI individuals according to medication response; therefore, our final dataset consisted of 12 HC, 15 TSC, and 18 EPI.

According to the Bristol Stool Form Scale (BSFC), none of the enrolled children were severely constipated or experiencing diarrhea.

### 3.2 Nutritional evaluation

Since diet is recognized as one of the key factors shaping the gut microbiota (Zmora et al., 2018), caregivers were asked to complete a 3-day food diary to assess the intake of micro- and macronutrients in the enrolled subjects.

The food diary analysis revealed a reduced daily energy intake in TSC subjects (TSC vs. HC, not significant; TSC vs. EPI, *p* = 0.005), although all values remained within the range recommended by Italian national guidelines (Italian Society of Human Nutrition (SINU), 2024). At the macronutrient level, no statistically significant differences were observed between the TSC and HC groups. However, the EPI group showed a higher intake of protein, lipids, and saturated fats (in grams) compared to the TSC group (*p* = 0.009, *p* = 0.004, and *p* = 0.038, respectively). Despite these findings, no significant differences were observed among the groups in protein, lipid, carbohydrate, or dietary fiber intake when macronutrients were assessed as a percentage of total energy intake. Macronutrient values - except for fats, which were elevated across all groups - aligned with Italian national recommendations [Italian Society of Human Nutrition (SINU), 2024]. A detailed table of diet evaluation is provided in the Supplementary Table S1.

### 3.3 Biodiversity assessment of gut microbial community

Microbiota profiling was performed through V3-V4 16S rRNA gene-targeted sequencing. After quality filtering processes, we obtained a mean count of 31,953 (+/− 5,840) reads per sample. Sequencing depth was set to the lowest sequenced sample (*n* = 18,216 reads), to compensate for the sequencing unevenness of the samples and to provide a consistent minimum amount for the downstream analysis.

**Figure 1:**
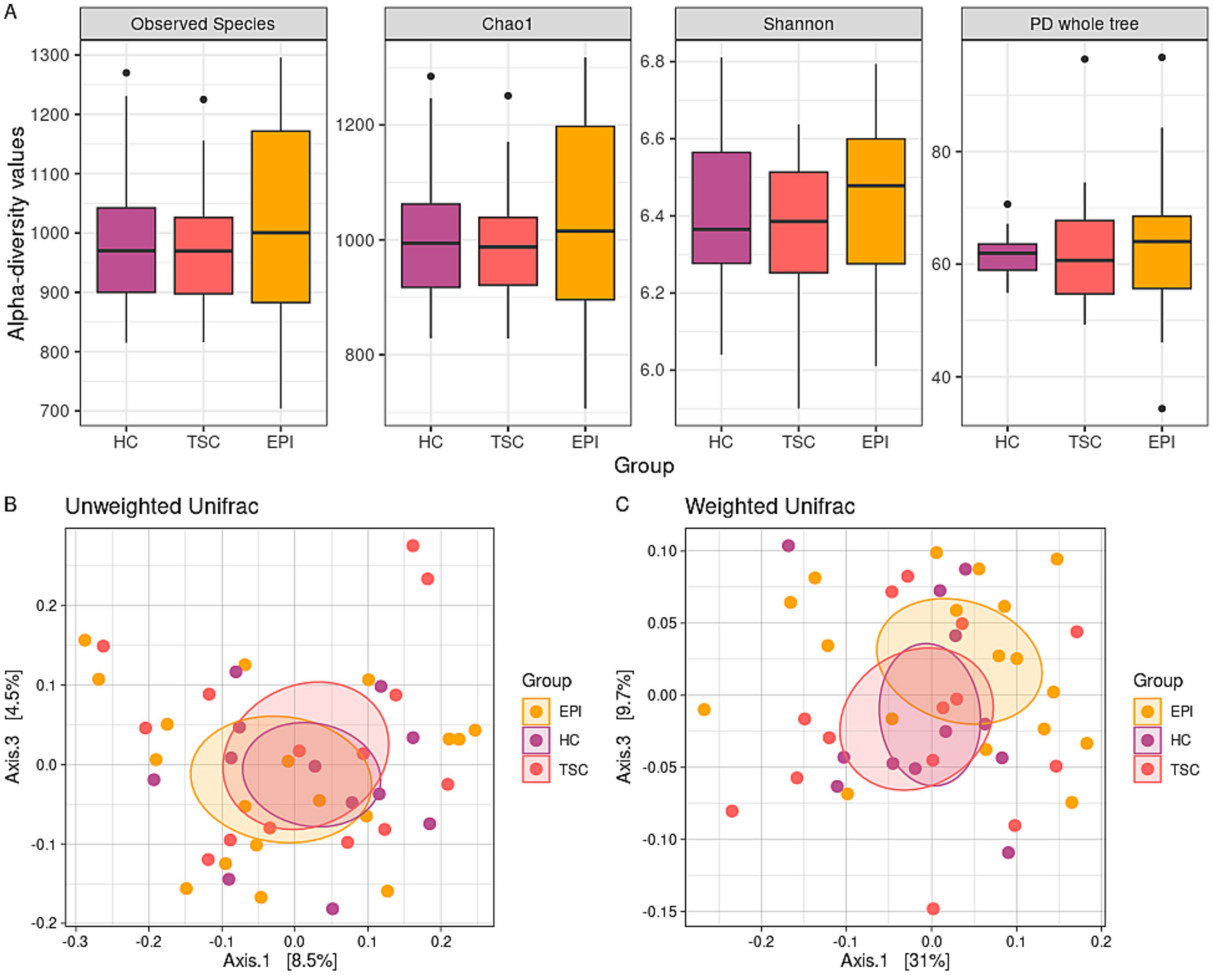
Microbiota biodiversity characterization. Panel **A**: box plots of alpha-diversity metrics across the three groups. No statistically significant differences were observed in the Observed Species (*p* = 0.820, *p* = 0.735, and *p* = 0.587 for HC vs. TSC, HC vs. EPI, TSC vs. EPI, respectively), Chao1 (*p* = 0.820 for all the analyses), Shannon (*p* = 0.820, *p* = 0.723, and *p* = 0.587), and PD Whole Tree (*p* = 0.781, *p* = 0.819, and *p* = 0.667) indexes. Panels **B,C**: principal coordinate analysis (PCoA) plots display beta-diversity among the three groups. Panel **B** depicts the unweighted Unifrac matrix of dissimilarity, while Panel **C** shows the weighted Unifrac metric. The first and third principal coordinates are reported for both measures. All comparisons between experimental groups were not significant.

Alpha-diversity analyses (Figure 1A) did not indicate significant differences between the HC, TSC, and EPI groups for both species richness and biodiversity.

Similarly, beta-diversity analysis (Figures 1B,C) fails to reveal significant differences in terms of microbiota dissimilarity between the cohorts. However, Weighted Unifrac distance (panel C) showed a trend toward distinct clusters between HC and TSD (raw *p*-value = 0.035, adj *p*-value = 0.105) and to a lesser extent between HC and EPI (raw *p*-value = 0.074, adj *p*-value = 0.111).

**Figure 2:**
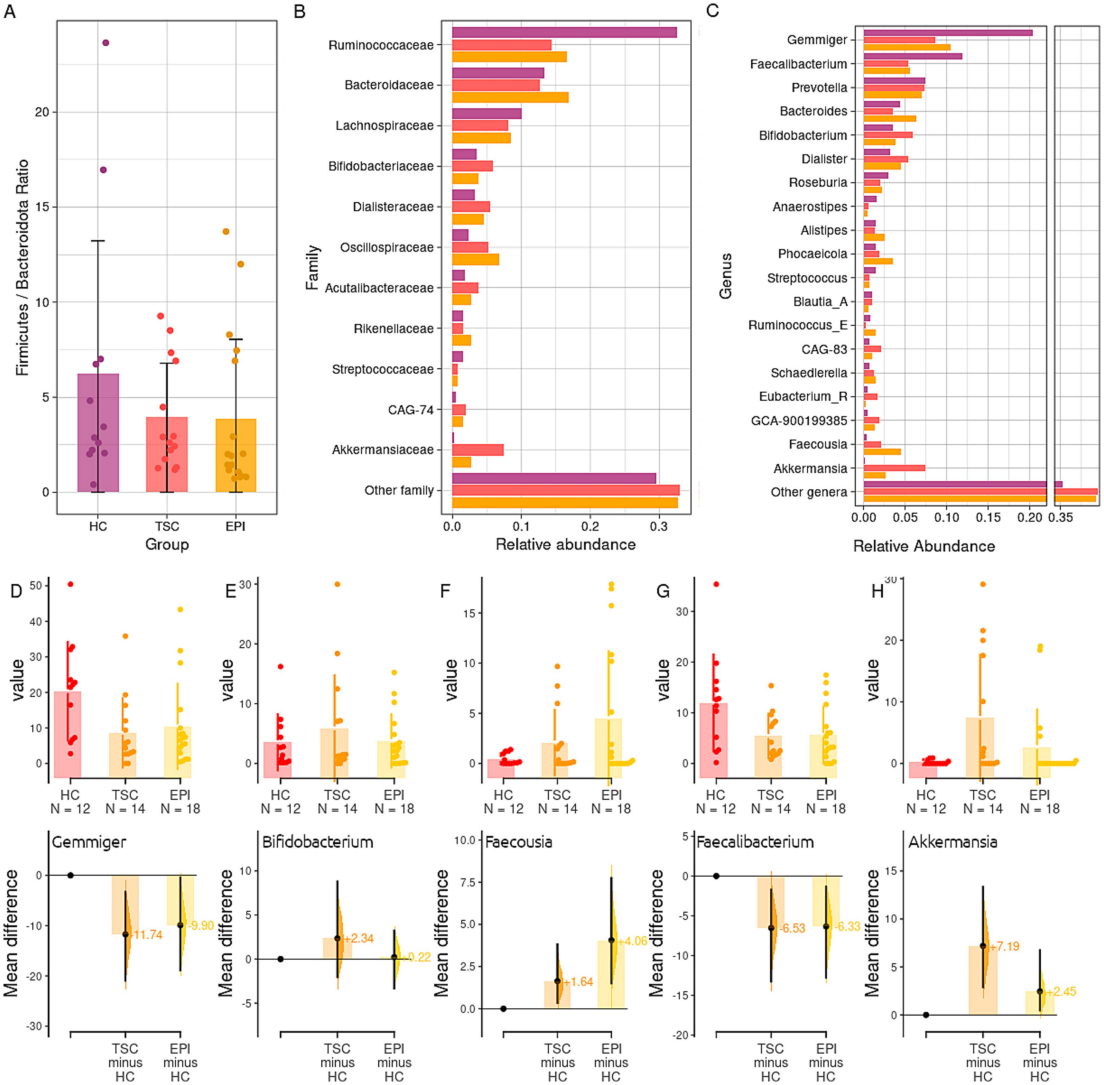
Taxonomy analysis. Panel **A**: boxplot of the ratio between the abundance of Bacteroidota and Firmicutes phyla in the three groups. Mean ratios (SD) are HC 6.23 (6.98), TSC 3.93 (2.84), EPI 3.85 (4.18). Panel **B**: Taxonomy analysis at family level of the gut microbiota in HC, TSC, and EPI groups. Panel **C**: relative abundance of bacteria genera. Only taxa with a mean relative abundance > 0.01 in at least one of the two experimental groups have been reported. See Supplementary Table S2 for the full list and statistics. Panel **D–H**: Gardner-Altman estimation plots for, respectively, the genera *Gemmiger* **(D)**, *Bifidobacterium* **(E)**, *Faecousia* **(F)**, *Faecalibacterium* **(G)**, *Akkermansia* **(H)**. The upper parts of the plots depict the groups’ abundances and distribution among the single samples; the lower parts report TSC and EPI average differences and effect sizes with respect to the HC group (set as 0).

When detailing the taxonomy phylogenetic levels among the three studied groups (Figure 2), we observed a significant decrease in the Firmicutes relative abundances within the TSC and EPI groups compared to healthy controls (50.1% HC vs. 33.6% TSC, adj *p* = 0.015; 50.1% HC vs. 38.0% EPI, adj *p* = 0.044). In agreement, the Firmicutes/Bacteroidota ratio was reduced in TSC and EPI groups (Figure 2A).

Among Firmicutes, the *Ruminococcaceae* family (Figure 2B) was found to be significantly reduced in both clinical groups compared to the HC subjects (32.6% HC vs. 16.6% EPI, *p* = 0.014; 32.6% HC vs. 16.1% TSC, *p* = 0.015). It is worth noticing that, although not significantly, the *Bacteroidaceae* family reported lower relative abundances within the HC group (13.3% in HC vs. 15.8% TSC and 16.9% EPI), while the *Oscillospiraceae* family was observed to be consistently higher in the EPI group (6.8% vs. 2.3% HC and 3.0% TSC). At the genus level (Figure 2C), we found HC microbial communities to be characterized by higher levels of *Gemmiger* (20.4% HC vs. 10.5% EPI, *p* = 0.043; 10.3% TSC, *p* = 0.073; Figure 2D), *Blautia_A* (1% HC vs. 0.6% EPI, *p* = 0.010) *Faecalibacterium* (11.9% HC vs. 5.5% TSC, *p* = 0.118; Figure 2G). Subjects with TSC, compared to the EPI, showed higher abundances of *Bifidobacterium* (Figure 2E), *Prevotella*, and *Akkermansia* (Figure 2H) spp. Subjects with EPI, on the other hand, had consistently higher abundance of *Bacteroides, Phocaeicola,* and *Faecousia* (Figure 2F). Taxonomy data is extensively detailed in Supplementary Table S2.

**Figure 3:**
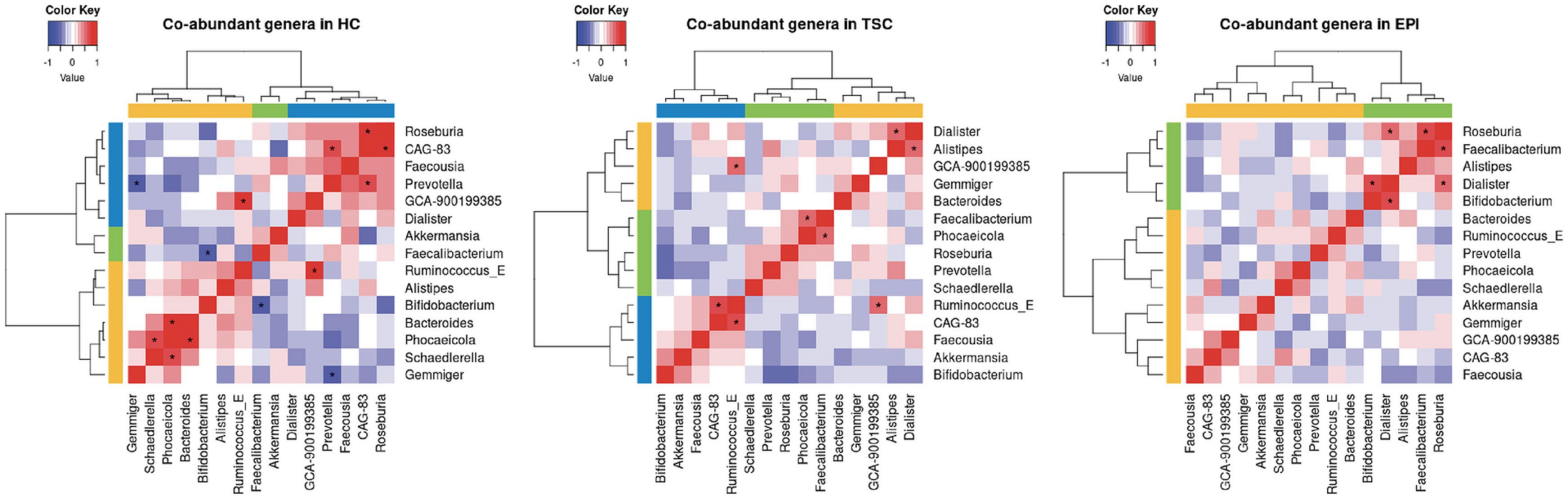
Co-abundance correlation analysis at genus level for HC, TSC, and EPI subjects. For each group, only taxa with relative abundance >0.01 are listed. Pearson’s correlation R values range from −1 (negative correlation, blue) to 1 (positive, red). Yellow, blue, and green hierarchical clusters represent different Co-Abundant Groups (CAGs). Asterisks (*) report statistical significance (adj *p* < 0.05).

Co-abundance relationships among the bacterial genera in HC, TSC, and EPI groups are reported in Figure 3. The progression from HC to EPI to TSC illustrates a gradient of microbial network disruption: while HC maintains robust and interconnected microbial communities, EPI patients exhibit moderate disruption, and pwTSC show significant fragmentation. The hierarchical cluster analysis identified three Co-Abundance Groups (CAGs) in HC and TSC, and two CAGs in the EPI group. All CAGs clustered differently between groups but showed comparable compositions. HC revealed two CAGs of bacteria positively related: one comprising the butyrate producers *Roseburia* and CAG-83 (belonging to the *Oscillospiraceae*), and the second dominated by Bacteroidia (*Bacteroides* and *Phoecaeicola*) and by *Gemmiger*, the most depleted taxon in both TSC and EPI. *Akkermansia* and *Faecalibacterium*, on the other hand, group together within a third HC CAG. TSC also presented three CAGs: one dominated by *Bifidobacterium* and *Akkermansia*, both enriched in TSC, plus *Faecousia* and *Ruminococcus*. This CAG is negatively related to the second, encompassing *Faecalibacterium* (strongly depleted)*, Roseburia*, *Phoecaeicola,* and *Prevotella,* while the third CAG comprises *Dialister, Alistipes*, *Bacteroides,* and *Gemmiger*. The EPI microbial community presented only two CAGs, of which only one was characterized by significant positive co-abundances that included both depleted, *Roseburia* and *Faecalibacterium,* and increased genera, i.e., *Alistipes*.

### 3.4 Microbial metabolite analysis

Changes in the relative abundance of microbial species, combined with a diet that influences substrate availability, can lead to variations in the production and release of microbial metabolites. Total SCFA content was similar in the three enrolled groups. In agreement, no significant differences were found in acetate (*p* = 0.453), propionate (*p* = 0.291), and butyrate (*p* = 0.902), as well as in the branched-chain fatty acids (BCFAs) Isobutyrate (*p* = 0.113) and Isovalerate (*p* = 0.064; see Supplementary Figure S1).

### 3.5 Diet-metabolomic-microbiome interactions

**Figure 4:**
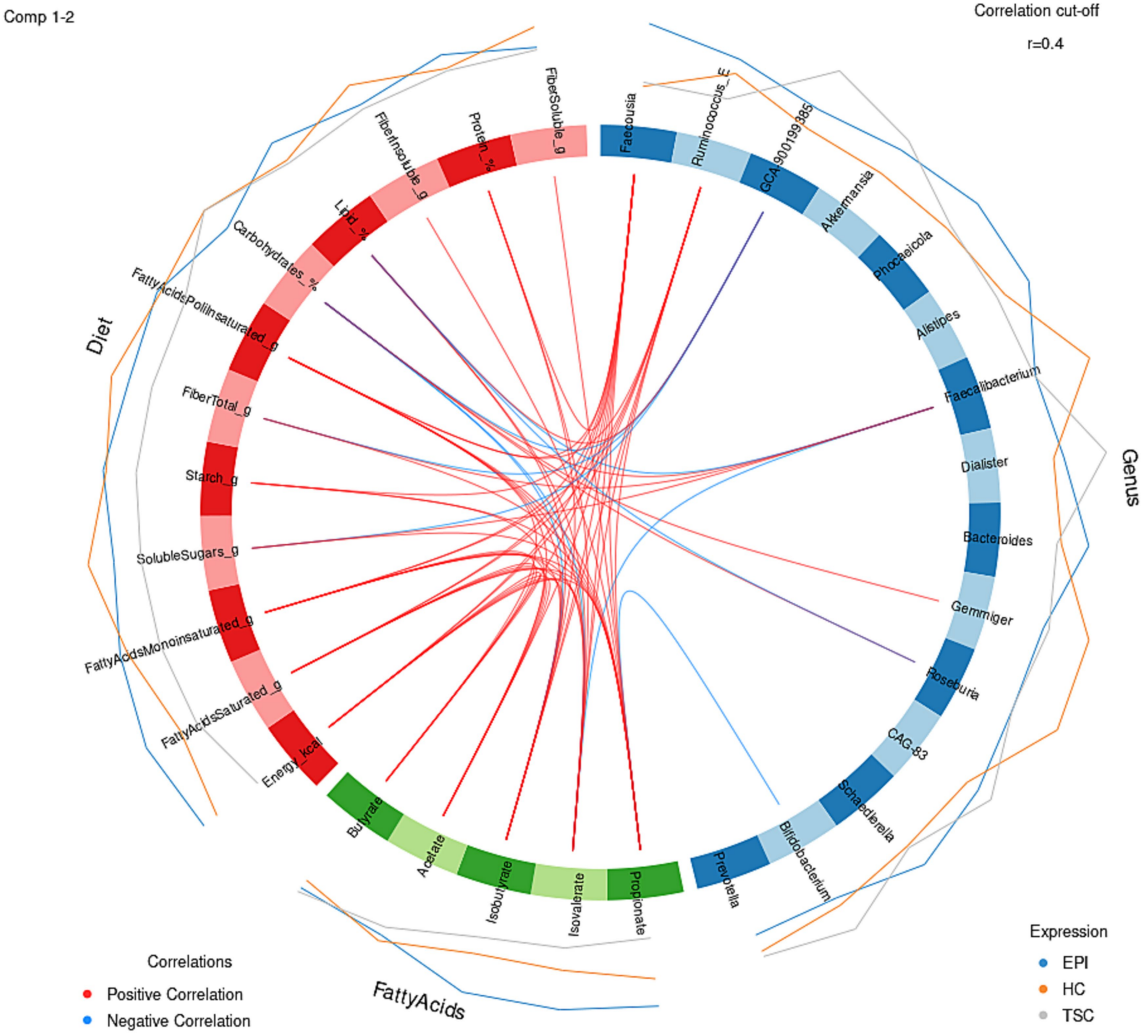
Circular plot for dietary macronutrient (red block), key metabolites (green block), microbial genera (blue block) data integration, with a |r| = 0.4 correlation cutoff. Positive associations are depicted with red lines, negative ones in light blue. External blue, gray, and orange lines represent the features’ expression in EPI, TSC, and HC, respectively.

To explore links among diet, gut microbiota, and microbial metabolites, we integrated diet–metabolite–microbiota data using sparse discriminant analysis (Figure 4). The block correlation analysis revealed key associations: *Faecalibacterium*, reduced in both EPI and TSC groups, correlated negatively with BCFAs and total lipids but positively with total fiber. Conversely, BCFAs were positively associated with *Faecousia* (enriched in EPI subjects) and protein intake, reflecting fermentation-derived production. *Bifidobacterium*, slightly increased in TSC in an individual-dependent manner, correlated negatively with propionate, which was lowest in TSC. *Gemmiger*, characteristic of the HC microbiota, correlated positively with carbohydrate intake.

## 4 Discussion

To our knowledge, this is the first study investigating the possible contribution of the gut microbial communities to the neurological features of TSC, especially regarding epilepsy.

In recent years, the gut microbiota has garnered increasing interest in the field of neuroscience, although its role although its role in epilepsy is still in its early stages, several promising findings have already emerged (Ceccarani et al., 2021; Riva et al., 2025; Zhu et al., 2024). Indeed, alterations in microbiota composition have been reported in individuals with epilepsy as well as in certain animal models (Zhu et al., 2024). Both preclinical and clinical studies suggest that modulating the gut microbiota may have antiseizure effects, highlighting its potential not only as a biomarker but also as a therapeutic target (Gong et al., 2021; He et al., 2017; Gómez-Eguílaz et al., 2018). For instance, recent studies demonstrated the beneficial effect of probiotics as an adjunctive treatment in drug-resistant epilepsy (Gómez-Eguílaz et al., 2018; El-Sharkawy et al., 2024).

TSC is associated with epilepsy in 70–90% (Curatolo et al., 2015; Vignoli et al., 2013) and can cause developmental epileptic encephalopathy due to early onset epilepsy and associated neurodevelopmental disorders (Jóźwiak et al., 2025) and undoubtedly linked to the genetic substrate underlying the disorder (Ng et al., 2022). Generally, patients carrying *TSC2* pathogenic variants, as the majority of the subjects enrolled in the present study, present a more severe phenotype, characterized by a higher number of tubers, earlier age at seizure onset, and higher prevalence of ID (Curatolo et al., 2023). Nevertheless, the clinical phenotype may show a high variability, and recent preclinical studies and human reports have suggested a possible role of inflammatory processes, particularly the activation of microglia, increased expression of pro-inflammatory cytokines, as well as aberrant mTOR-mediated immune responses, in the development and progression of neurological symptoms in pwTSC (Kaur et al., 2021; Xie et al., 2020; Fuso et al., 2016; Balthazard et al., 2025; Gruber et al., 2022).

A recent study in a *Tsc2^+/−^* mouse model showed that deficiency of the *TSC2* gene causes different gut microenvironments, which may be linked to decreased connectivity and sociability. Furthermore, after a treatment with dietary curcumin, the abundance of certain bacterial taxa was greatly increasedand corresponded to increased myelination and white matter plasticity, contributing to improved sociability in *Tsc2^+/−^* mice (Hsieh et al., 2024).

In this study, we characterized the gut microbiota of individuals with TSC and compared it to age- and sex-matched neurotypical controls and individuals with non-TSC epilepsy. A dietary survey was conducted to control for environmental influences, revealing no major differences among groups. Likewise, SCFA and BCFA levels did not differ significantly between groups.

Alpha-diversity metrics, which reflect the biodiversity within each sample, revealed no significant differences in species evenness or richness among the study groups. Regarding beta-diversity, although the differences did not reach statistical significance, we observed a trend toward clustering of TSC and EPI individuals, distinct from HC, consistent with existing literature suggesting an epilepsy-associated gut dysbiosis (Peng et al., 2018).

The taxonomic analysis highlighted a depletion of Firmicutes in both TSC and EPI, resulting in a decrease of F/B ratio, in agreement with literature studies (Zhu et al., 2024; Ceccarani et al., 2021). In our cohort, the observed decrease in Firmicutes appears to be primarily driven by a reduction in the *Ruminococcaceae* family, and at the genus level, by *Gemmiger*, and to a lesser extent, *Faecalibacterium* spp. *Gemmiger* has recently been identified as a biomarker of a healthy gut microbiota, noted for its anti-inflammatory properties. Together with *Faecalibacterium* and *Roseburia*, *Gemmiger* defines the three co-abundance groups (CAGs) identified in the healthy control group and considered beneficial due to their health-promoting activities (Forbes et al., 2018; Borghi et al., 2024), primarily through the production of SCFAs (Kircher et al., 2022). Notably, all three taxa are capable of producing butyrate - a metabolite known for its wide-ranging positive effects - including the ability to mitigate epileptogenic stimuli in rodent models by reducing oxidative stress and neuroinflammation (Adebayo et al., 2025; Li et al., 2021).

Recently published research demonstrated that active epilepsy in individuals with TSC is associated with elevated levels of GFAP compared to those with TSC but without epilepsy. This finding was confirmed in an external validation cohort and was also accompanied by increased levels of pro-inflammatory cytokines, including IL-17A, IL-17C, and TNF-*α* (Peng et al., 2018). Different microbial taxa in the gut exert either pro-inflammatory or anti-inflammatory effects and have been reported to modulate both local and systemic inflammation. Notably, *Faecalibacterium* and *Roseburia* are the most frequently reported for dampening the inflammation (Zhu et al., 2024). Their ability to modulate the Th17/Treg balance toward a more tolerogenic profile relies on their production of butyrate and its histone deacetylase (HDAC) inhibitory activity (Zhou et al., 2018).

Most of the alterations described in this exploratory study, including the above-mentioned depletion in *Gemmiger* and *Faecalibacterium*, were shared between TSC and EPI groups, suggesting that epilepsy may be the common underlying factor. However, some distinctions were noted: the TSC group exhibited an enrichment of *Akkermansiaceae* compared to both HC and EPI groups, while the EPI group showed a decreased relative abundance of *Blautia_A* and *Faecousia*. Increased abundance of *Akkermansia,* which to a lesser extent also involves the EPI group, has been observed in other neurological disorders, including epilepsy itself (Ceccarani et al., 2021), multiple sclerosis (Jangi et al., 2016), Alzheimer’s disease, and Parkinson’s disease (Fang et al., 2021). In the TSC group, *Akkermansia* was positively related to *Bifidobacterium*, one of the CAGs. In contrast, in the HC group, *Akkermansia* was associated with *Faecalibacterium*, suggesting potential alterations in the microbial network dynamics between taxa.

We observed a significant reduction of the genus *Blautia* in the EPI group compared with HC. *Blautia* includes species with diverse metabolic properties and, in turn, effects on human health (Liu et al., 2021), but the V3–V4 sequencing of the 16S rRNA gene does not allow for precise identification of the depleted species, underestimating the potential role in epilepsy. The EPI group was characterized by an enrichment of *Faecousia*, a recently described taxon belonging to the *Oscillospiraceae* family, with predicted capabilities for starch utilization and production of SCFAs (Hitch et al., 2025). Although *Oscillospiraceae* is generally considered a beneficial family, the significance of this finding remains difficult to interpret given the limited current knowledge (Yang et al., 2021).

Considering the whole spectrum of TANDs, many individuals with TSC in our cohort showed ID and/or neurodevelopmental disorder (ASD or ADHD). ASD is characterized by a distinct intestinal bacterial signature, and neuroinflammation has been proposed as an underlying mechanism. Indeed, increased intestinal permeability may pave the way to neuroinflammation via cytokines, leading to synaptic dysfunction and failure of microglia maturation (Hsiao et al., 2013). The bacterial phyla most frequently associated with higher inflammatory cytokine levels in ASD children are *Prevotella, Bacteroidetes,* and *Bifidobacterium* (Morton et al., 2023). Intriguingly, these genera were also enriched in our TSC group compared to HC and EPI, highlighting shared microbial signatures potentially associated with specific clinical features.

## 5 Limitations and future directions

These preliminary findings, although derived from a small cohort, provide an important first step in clarifying the role of the microbiota–gut–brain axis (MGBA) in TSC. Larger, multi-center studies will be essential to confirm these results and to enable subgroup analyses based on epilepsy-related factors (e.g., duration, type and number of antiseizure medications) as well as neuropsychiatric profiles, which may yield more nuanced insights.

The sample size, while sufficient to identify broad trends, may limit the detection of more subtle associations. To ensure transparency, results close to conventional significance thresholds are reported with exact *p*-values and descriptive statistics.

Methodologically, the use of V3-V4 16S rRNA sequencing provides valuable taxonomic insight but restricts resolution at the genus level and does not capture microbial functional activity.

The gut microbiota plays a powerful role in shaping inflammation, which through the gut-brain axis may fuel epileptogenesis and worsen neurological symptoms in TSC. Given that the gut microbiota is both accessible and modifiable, investigating its potential role could offer promising avenues for the development of more personalized and effective treatments. Future studies with larger cohorts, longitudinal sampling, and multi-omics approaches will be necessary to confirm and expand upon these preliminary findings.

## Data Availability

The 16S rRNA gene sequences obtained from this study were deposited in the NCBI Short-reads Archive (SRA) repository with BioProject accession number PRJNA1269281 (https://www.ncbi.nlm.nih.gov/sra/).
