# Correction: Gut microbiota signatures in tuberous sclerosis complex and epilepsy: a pilot study

**DOI:** 10.3389/fnins.2025.1760688

**Published:** 2026-01-16

**Authors:** Emerenziana Ottaviano, Matteo Domenico Marsiglia, Camilla Ceccarani, Silvia Ancona, Francesca Triva, Francesca La Briola, Stefania Bergamoni, Federica Teutonico, Alice Pompili, Ilaria Viganò, Emilia Ricci, Aglaia Vignoli, Elisa Borghi

**Affiliations:** 1 Department of Health Sciences, Università Degli Studi di Milano, Milan, Italy; 2 Institute of Biomedical Technologies, National Research Council, Milan, Italy; 3 Child Neurology and Epilepsy Centre, ASST Santi Paolo e Carlo, Milan, Italy; 4 Childhood and Adolescence Neurology and Psychiatry Unit, ASST GOM Niguarda, Milan, Italy; 5 Università Degli Studi di Milano, Milan, Italy

**Keywords:** tuberous sclerosis complex, gut microbiota brain axis, epilepsy, inflammation, children

Author Alice Pompili was erroneously assigned to affiliation “4 Childhood and Adolescence Neurology and Psychiatry Unit, ASST GOM Niguarda, Milan, Italy”. This affiliation has now been removed for author Alice Pompili. Affiliation “5 Università Degli Studi di Milano, Milan, Italy” was omitted from the paper. This affiliation has now been added for author Alice Pompili.

The title of this article was erroneously given as: Tuberous sclerosis complex, epilepsy, and the microbiota-gut-brain axis: a pilot study of shared and divergent microbial signatures.

The correct title of the article is **Gut microbiota signatures in tuberous sclerosis complex and epilepsy: a pilot study**.

There was a mistake in **Figures 3** and **4** as published. **Figures 3** and **4** were inadvertently swapped, while the corresponding legends remain correct.

The original version of this article has been updated.

