# Supplementary figures and images for: Gut microbiota signatures in tuberous sclerosis complex and epilepsy: a pilot study
Source: Front Neurosci. 2025 Nov 18;19:1655456. doi: 10.3389/fnins.2025.1655456 (PMC12670250; doi:10.3389/fnins.2025.1655456)

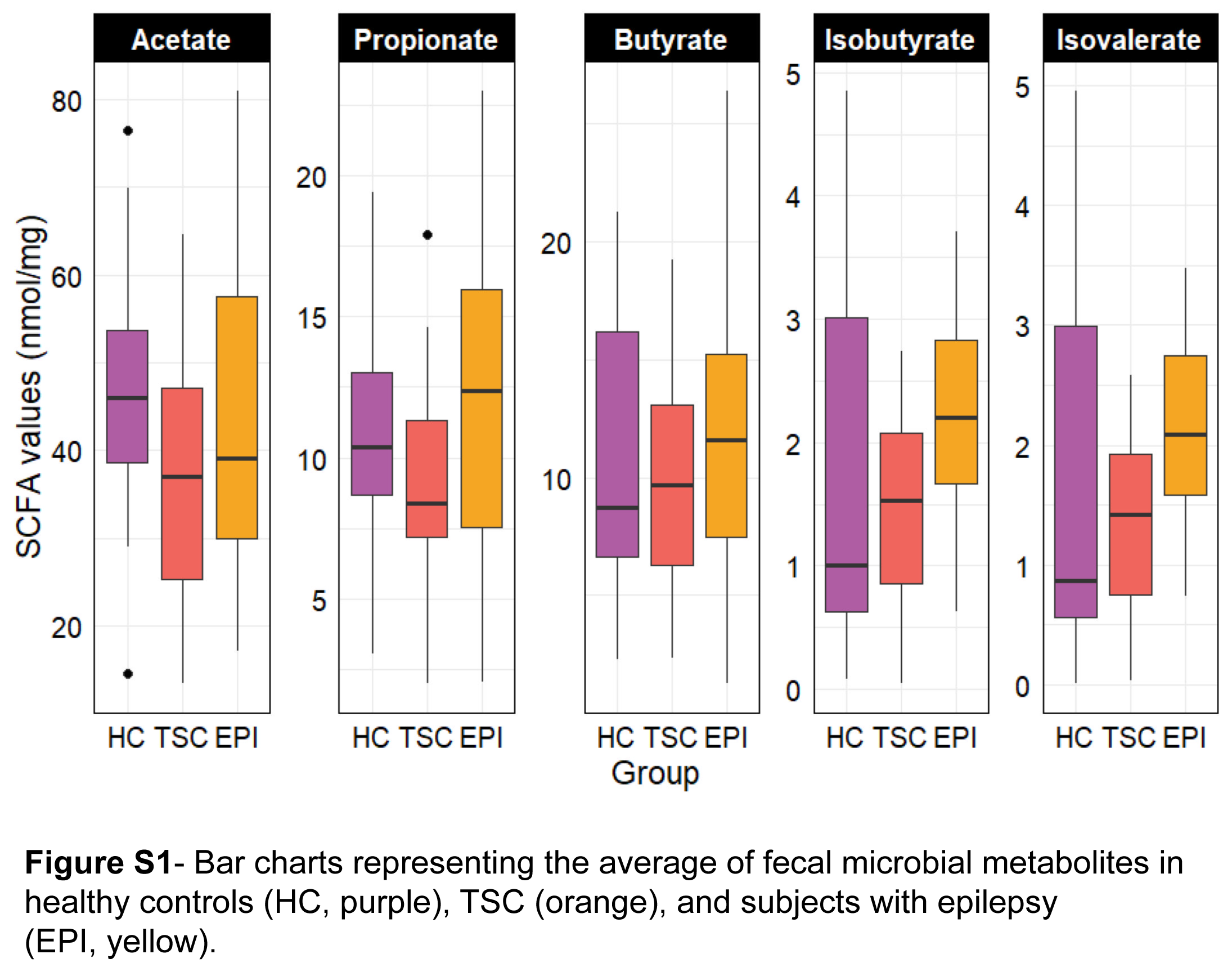

Supplement: Supplementary file 1 [file Image_1.TIF]
