# Supplementary material for: Gut microbiota signatures in tuberous sclerosis complex and epilepsy: a pilot study
Source: Front Neurosci. 2025 Nov 18;19:1655456. doi: 10.3389/fnins.2025.1655456 (PMC12670250; doi:10.3389/fnins.2025.1655456)
Supplement: Supplementary file 2 [file Table_1.DOCX]

|  | **HC Mean ± SD** | **TSC Mean ± SD** | **EPI Mean ± SD** | ***p*-value** | **Reference values**  **(LARN, pediatrics)^°°^** | **Reference values**  **(LARN, adults)^°°^** |
| --- | --- | --- | --- | --- | --- | --- |
| Energy (En) kcal | 1534.2 ± 332.2 | 1230.3 ± 281.5 | 1658.9 ± 379.9 | 0.005 (TSC vs EPI) | 710 - 1830 kcal (BMR kcal/die*) | 1237- 2050 kcal  (BMR kcal/die*) |
| Proteins g | 57.9 ± 14.7 | 46.6 ± 16.3 | 67.4 ± 21.1 | 0.009 (TSC vs EPI) | 9–50 (g/die) (AR) | 43-50 (g/die) |
| Proteins %E | 15.5 ± 2.8 | 15.3 ± 3.1 | 16.4 ± 2.7 | ns^§^ | 0.72-0.82 (g/kg×die) (AR) | 0.71 % (AR) |
| Total Carbohydrates g | 196.4 ± 52.4 | 150.5 ± 29.6 | 195.1 ± 70.7 | ns |  |  |
| Total Carbohydrates %E | 48.4 ± 3.9 | 47.5 ± 5.6 | 44.0 ± 10.3 | ns | 45%–60% En | 45%–60% En |
| Sugars g | 65.2 ± 19.8 | 46.7 ± 18.2 | 56.2 ± 23.1 | ns | <15% En |  |
| Starch g | 82.1 ± 39.7 | 56.1 ± 22.8 | 86.7 ± 51.9 | ns |  |  |
| Fiber g | 13.7 ± 4.2 | 10.9 ± 4.0 | 12.2 ± 4.6 | ns | 8.4 g/1000 Kcal | 12.6–16.7  g/1000 Kcal% |
| Soluble Fiber g | 2.0 ± 1.2 | 1.5 ± 0.7 | 1.7 ± 0.9 | ns |  |  |
| Insoluble Fibers g | 6.1 ± 4.0 | 4.5 ± 2.0 | 4.9 ± 2.8 | ns |  |  |
| Fats g | 60.7 ± 10.4 | 50.1 ± 14.6 | 70.2 ± 15.5 | 0.004 (TSC vs EPI) |  |  |
| Fats %E | 36.7 ± 3.8 | 37.1 ± 3.8 | 39.5 ± 9.3 | ns | 20%–35% En | 20%–35% En |
| Saturated Fats g | 18.4 ± 5.0 | 16.7 ± 4.5 | 23.7 ± 6.9 | 0.038 (TSC vs EPI) | <10% En | <10% En |

**Table S1**- Daily dietary intake of energy and macronutrients in healthy controls (HC), and individuals with tuberous sclerosis complex (TSC) and epilepsy (EPI).

p-values <0.05 are considered significant (Kruskal-Wallis test). ^°°^LARN, Nutrients and Energy Reference Intake Levels for the Italian Population; ^§^ns, non-significant; *daily energy required to maintain basal metabolic rate (BMR).
