# Supplementary material for: Gut microbiota signatures in tuberous sclerosis complex and epilepsy: a pilot study
Source: Front Neurosci. 2025 Nov 18;19:1655456. doi: 10.3389/fnins.2025.1655456 (PMC12670250; doi:10.3389/fnins.2025.1655456)
Supplement: Supplementary file 3 [file Table_2.DOCX]

**Table S2** - Gut microbiota composition in in healthy controls (HC), and individuals with tuberous sclerosis complex (TSC) and epilepsy (EPI).


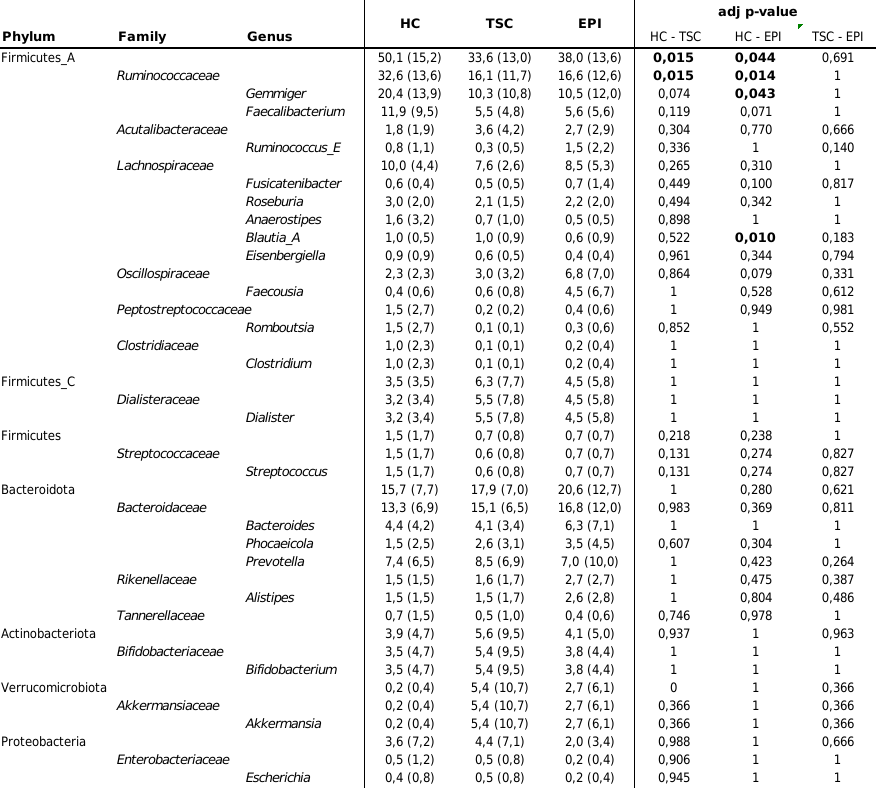


Major bacterial groups were organized in three phylogenetic levels (phylum, family, genus) and reported as average relative abundance ± standard deviation. p-values <0.05 were considered significant.
